# The COVID-19 pandemic as disjuncture: Lifelong learning in a context of fear

**DOI:** 10.1007/s11159-020-09863-w

**Published:** 2020-10-30

**Authors:** Cecilia Bjursell

**Affiliations:** National Centre for Lifelong Learning, School of Education and Communication, Jönköping University, Jönköping, Sweden

**Keywords:** disjuncture, pandemic, lifelong learning, social distancing, intergenerational relationships

## Abstract

The COVID-19 pandemic has caused a number of fundamental changes in different societies, and can therefore be understood as creating “disjuncture” in our lives. *Disjuncture* is a concept proposed by adult educator Peter Jarvis to describe the phenomenon of what happens when an individual is confronted with an experience that conflicts with her/his previous understanding of the world. Faced with a situation that creates disjuncture, the person is compelled to find new knowledge and new ways of doing things; i.e., he/she must embark on a learning process. The recent introduction of *social distancing* as a measure aiming to reduce transmission of the COVID-19 virus has dramatically changed people’s behaviour, but this measure does not only have preventive and desirable effects. There is an associated risk for *increased isolation* among the older generations of the population, as well as a *change in intergenerational relationships*. Although the current pandemic (as disjuncture) may potentially initiate major learning processes in the human collective, we should remember that disjuncture is often theorised within neutral, or even positive, contexts. In a context of fear, however, learning may result in a narrowing of mindsets and a rejection of collective efforts and solidarity between generations. In terms of the types of learning triggered by the current pandemic (as disjuncture), one problem is *non-reflective learning,* which primarily occurs on a behavioural level. We need to recognise this and engage in *reflective learning* if we are to make the choices that will lead to a society that is worth living in for all generations. Our goal must be to learn to be a person in a post-pandemic society.

## Résumé

La pandémie de COVID-19, une disjonction : l’apprentissage tout au long de la vie dans un contexte de peur – la pandémie de COVID-19 a provoqué un ensemble de changements fondamentaux dans différentes sociétés et l’on peut, par conséquent, en déduire qu’elle a créé une « disjonction » dans nos existences. La *disjonction* est un concept proposé par l’éducateur d’adultes Peter Jarvis pour décrire le phénomène survenant quand *un individu* est confronté à une expérience contradictoire avec sa perception antérieure du monde. Face à une situation qui crée une disjonction, la personne est forcée d’acquérir de nouvelles connaissances et de trouver de nouvelles façons de faire les choses : ainsi doit-elle, par exemple, entreprendre un processus d’apprentissage. Si la récente introduction de la *distanciation sociale*, une mesure qui vise à réduire la transmission de la COVID-19, a entraîné un changement spectaculaire dans le comportement des gens, elle n’a toutefois pas eu uniquement des effets préventifs et désirables. Elle est en effet associée à un risque *d’isolement accru* pour les générations âgées de la population ainsi qu’à une *mutation des rapports intergénérationnels*. Bien que l’actuelle pandémie (en tant que disjonction) puisse potentiellement déclencher d’importants processus d’apprentissage au sein du collectif humain, nous devons garder présent à l’esprit que la disjonction est souvent théorisée dans des contextes neutres, voire positifs. Dans un contexte de peur, néanmoins, l’apprentissage pourrait avoir pour conséquence de rendre les mentalités plus fermées et de rejeter les efforts collectifs et la solidarité entre les générations. En ce qui concerne les types d’apprentissages déclenchés par l’actuelle pandémie (en tant que disjonction), on constate un problème qui apparaît d’abord sur le plan comportemental : *l’apprentissage non réflexif.* Nous devons reconnaître cela et nous livrer à un *apprentissage réflexif* si nous voulons faire les choix qui conduiront à créer une société où il vaut la peine de vivre pour toutes les générations. Notre objectif doit consister à apprendre à devenir des personnes dans une société postpandémique.

## Introduction

The COVID-19 pandemic has created a major “disjuncture” in our existence. Within a short period of time, our lives have been turned upside down, in a way no one could have imagined. On 11 March 2020, the World Health Organization (WHO) officially classified the spread of a novel coronavirus, SARS-CoV-2, as a *pandemic*,^1^ prompting many communities to act by closing their borders and imposing curfews on the movement of people. To reduce the spread of infection, national governments recommended that their citizens should engage in “social distancing”. *Social distancing* means keeping a *physical* distance (in the current pandemic, the stipulated space varies between 1.5 to 2 metres) from other human beings.^2^ The term refers to measures that people can/should take to reduce personal proximity, an integral part of their social interaction with other people, and thus help reduce the transmission of an infectious disease such as the coronavirus. These directives to change what was hitherto entirely “normal” behaviour among human beings has caused “disjuncture” in people’s lives; namely, a disharmony between the world as we knew it and the state of the world during the current pandemic.

*Disjuncture* is a concept proposed by adult educator Peter Jarvis to describe the phenomenon of what happens when an individual is confronted with an experience that conflicts with her/his previous understanding of the world.

Our lives comprise a series of experiences made up in such a way that our biography appears as continuous experience added to by each unique episode of learning which we call an experience. But each of these unique episodes begins with the same type of question […]: Why has this occurred? How do I do this? What does this mean? And so on. It can be cognitive, emotional or a combination of the two: it is this that I call disjuncture (Jarvis 2012a, p. 76).

According to *lifelong learning*^3^ theory, disjuncture triggers learning, but what is it that we learn during a pandemic? Italian philosopher Giorgio Agamben argues that panic has paralysed his country (Italy) and that people have

sacrifice[d] practically everything – the normal conditions of life, social relationships, work, even friendships, affections, and religious and political convictions – to the danger of getting sick (Agamben 2020).

Fear and panic can have serious long-term outcomes for individuals and society, including the worsening of existing problems, such as isolation among the older generations of the population and the gap between generations. It therefore seems likely that an examination of the changes that occur during a pandemic will be key to understanding the learning that takes place in a context of fear. As this article is being written during an ongoing pandemic, readers should bear in mind that the situation may change dramatically and we do not know how this might impact people’s learning at different points in time. The discussion in this paper is intended to inform and stimulate debate and further research.

The purpose of this article is to revisit the concept of *disjuncture* (Jarvis 2012a) and to invoke lifelong learning theories because they have the potential to: (1) allow us to conceptualise the current pandemic as a learning process; (2) connect the practice of social distancing to ongoing, long-term changes in society; and (3) highlight certain risks and possibilities which need to be addressed if our goal is to support people’s engagement in the kind of learning that is directed towards achieving a better post-pandemic life and a better post-pandemic society.

## Lifelong learning as policy and as a philosophy of education

### Lifelong learning as a policy

When lifelong learning became a central concept in global policy half a century ago, its proponents highlighted that: (1) learning is not only about child development, but continues throughout adult life; and (2) learning takes place in *every* context, not just within the confines of the school classroom. One of the seminal reports marking the establishment of lifelong learning as a concept was *Learning to be: The world of education today and tomorrow* (Faure et al. 1972).^4^ Often referred to as “the Faure report”, it emphasised a humanist, holistic vision of education that was applicable to all members of society.

Since the release of this report, lifelong learning policies have addressed social problems on a global scale in conjunction with the emergence of key institutions in this field. Moosung Lee and Tom Friedrich (2011) discuss differences in underlying ideology between the United Nations Educational, Scientific and Cultural Organization (UNESCO) on the one hand, and the Organisation for Economic Cooperation and Development (OECD), the European Union (EU), and the World Bank on the other. While UNESCO has had its own unique impact on international discussions, contemporary ideologies with respect to lifelong learning advanced by the other three organisations have been dominated by a neoliberal capitalist perspective (ibid.). This is unfortunate, because UNESCO’s enlightenment tradition (which is based on rationalism, progress, freedom, emancipation, and the concept of human beings as masters of their own destiny) is at odds with, and is actually undermined by, the utilitarian view of education that comes with adopting a neoliberal capitalist perspective (Elfert 2015).

This conflict is of particular interest during a pandemic such as the one we are currently experiencing, because many people will lose or have already lost their jobs, which is likely to strengthen the utilitarian view of education even more. Maren Elfert (ibid.) argues that the adoption of such an instrumental approach presents an obstacle to placing education in a wider societal context for those who aim to solve social problems on a global scale. Instead of reducing the process of education to the mere creation of productive economic units, the purpose of education should be to offer learning opportunities which foster the development of people who think critically, but not cynically, which then enables them to act as responsible citizens (Stanistreet 2020). One effort in particular that should be made to effectively address social problems, namely, the promotion of an intergenerational society, is discussed later in this article.

The concept of *lifelong learning* may lead to some confusion because in addition to being a core policy concept, it can also be invoked to refer both to an individual’s spectrum of learning and to institutions which offer educational opportunities (Jarvis 2004, 2014). A singular focus on institutions which offer formal education is too narrow, because learning also takes place in professional settings, work environments, and in groups and gatherings of various kinds. It is therefore impossible to create a fully *institutionalised* system of lifelong learning; irrespective of where learning takes place, such learning must be recognised as part of the person’s *total learning*.

A shift in terminology from *education* to *learning* entails a shift in meaning and focus. It might be said that education gives the active part to the teacher, while learning represents the active learner’s perspective. We thus observe a shift in focus from formal education to how an individual creates and transforms experiences into knowledge, skills, attitudes and values at every age, throughout this individual’s life.

Another drawback that can be associated with an instrumental/utilitarian approach to education is that it excludes the possibility of adopting a holistic understanding of human learning. If one is to understand learning as something which grows out of the experience of living, *being* and *becoming,*^5^ this demands that one understands that the self is shaped through thinking and doing over time, and is embedded in overlapping and contradictory *life-worlds*^6^ (Jarvis 2009, 2012a).

### Lifelong learning as a philosophy of education

As a philosophy of education, lifelong learning awards the individual’s learning a central position and highlights the fact that this must be the starting point if we are to properly understand what learning is about. Learning must be understood in its entirety and as the result of interaction between the individual and the individual’s environment. It is important to note that it is the learner who enters situations that provide experiences by which learning takes place (Jarvis 2007). Jarvis emphasises the point that, by starting with the learner (and not with what is being learned) we are led to the understanding that the learner’s biography is changed as a result of learning, irrespective of the person’s age. When experience is processed, it is integrated into the person’s being. This entails that a person experiences *being* and *becoming* in a lifelong process. Jarvis opposes the dualistic view of the division between body and soul because, he claims, it is impossible to separate one from the other (Jarvis 2009). To ground learning in the framework of being, rather than in a framework of having, entails that one accepts fluidity and dynamic movement as one strives to grasp one’s manner of existing (Su 2011). To think of learning as the mere acquisition of knowledge is, therefore, insufficient.

Learning takes place by means of lived relationships in a cultural context, as instantiated by a particular society. Each individual person’s process of shaping their identity is key to learning, because lifelong learning involves the continued development of a new understanding of “the self”, something which also entails a re-negotiation of one’s identity. During the current pandemic, many activities have moved online, including, for example, adult education. Even though teaching as such takes place in much the same manner as previously, this move to a digital environment entails a great deal of change and demands new competencies from teachers. These changes are so substantial that they give rise to questions concerning the individual’s perception of their self and their professional performance as teachers. This shift from the classroom to a digital environment via the internet is not just a practical issue; it also entails a shift from one state of existence to another.

In a similar manner, the implementation of a lockdown and social distancing creates a state of existence which prompts people to reflect on their selves and their situation. This can influence their perceptions of themselves and others. A pandemic such as the one we are currently experiencing influences individual people’s lives as well as the behaviour displayed by society as a whole. According to the terminology used in the context of lifelong learning as a philosophy of education, we thus conclude that a pandemic causes a *disjuncture*.

## Disjuncture and lifelong learning

*Disjuncture* has meanwhile become an established concept in lifelong learning which is used to explain a condition that enables learning. Peter Jarvis (2012b) proposed that learning in our everyday lives is often triggered by a gap between our expectations and an experience, i.e. disjuncture. Disjuncture refers to a state of disequilibrium, where we feel uneasy, out of our depth. In response to this feeling, we seek change – in order to achieve equilibrium and stability again. The process of striving towards a new state of equilibrium triggers the learning that takes place:

Indeed, this state of disequilibrium is a fundamental cause of learning that is inextricably intertwined with being-in-the-world: it is a part of the human condition (Jarvis 2016 [2001], p. 30).

Disjuncture, the gap between what we know and what we experience, provokes within us our initiative to understand and deal with particular situations, so that we can return to a harmonious state (Jarvis 2012b), albeit a new one. In other words, the need to learn is a fundamental need. As mentioned above, our learning takes place in interaction with our physical and social surroundings. Learning can be said to be the bridge that links the self to the world. When disjuncture occurs, a person may perceive a sense of detachment from the former self, because it no longer fits the new situation. Learning is the activity that enables a person to deal with disharmony and involves the movement from one state of being to another. This transition from one state to another requires that learning reformats a person’s previous knowledge and experience in relation to the new experience in a manner that could not be understood (by the individual) in terms of previously existing frameworks. Eventually, the individual will initiate a concluding phase of the learning process when new knowledge is incorporated into her/his state of being and new frameworks emerge. By means of the process thus described, we say that the individual has learned something. This could also be regarded as the end of the learning process initiated by the disjuncture.

However, it is in fact also part of the *continuous flow* of learning that takes place throughout a person’s life and thus sets a new direction for future learning. In his book on *Learning in later life,* Peter Jarvis notes that

it is recognized here that more than ever before in the history of humankind we have the opportunity to create our own biography through the choices that we make and we recognize that learning is the force through which our biographies develop and expand (Jarvis 2016 [2001], p. 45).

Being (and learning) always occurs in relation to a social situation. Jarvis states that being is driven by the moral imperative about achieving potential as human beings for the community in which we live (Jarvis 2012b). Engagement in the social world cannot be mastered by instinct. Instead, it has to be mastered by learning if we are to obtain the knowledge that is needed to cope with living in the world. A pandemic such as the one we are currently experiencing presents a new social situation, and we need the process of learning to deal with the situation.

However, there is also a risk that learning will not take place. With respect to an analysis of the effects of a pandemic as disjuncture, a typology of learning and non-learning is, therefore, of some help. The analysis I present below is guided by three categories of responses to new experiences, namely: (1) *non-learning*; (2) *non-reflective learning*; and (3) *reflective learning* (Jarvis 2016 [2001]).

Within each category, there are three types of learning or non-learning (Table 1). First, the three types of non-learning are *presumption*, *non-consideration* and *rejection*. The three different types of non-learning all refer to the fact that people do not always learn from their experiences. “Presumption” is a typical response to an experience that is familiar to the individual and the individual knows what to do, and therefore, there is no need for change. Non-learning can also occur when a person experiences something that could be a potential learning experience, but either because they do not understand the situation or they are not conscious of the situation (“non-consideration”) learning fails to take place. In addition, non-learning may be the case in situations where the person is aware of the potential for learning but they reject this possibility (hence the label “rejection”).

**Table 1:** Typology of learning responses to new experiences

Non-learning	Non-reflective learning	Reflective learning
Presumption	Preconscious learning	Contemplation
Non-consideration	Skills learning	Reflective skills learning
Rejection	Memorisation	Experimental learning

Based on Jarvis (2016 [2001])

The three types of non-reflective learning include *preconscious learning*, *skills learning* and *memorisation*. “Preconscious learning” takes place when an individual monitors their actions on a low level of consciousness, such as when driving a car. “Skills learning” occurs when skills are acquired in action through imitation. “Memorisation” is the process of verbal imitation, for example, when a person memorises the words of the instructor so as to be able to reproduce them at a later time. Non-reflective learning represents processes associated with social reproduction. These processes are commonly on the level of bodily experience, in contrast to the level of communicative interaction.

Lastly, the three types of reflective learning are *contemplation*, *reflective skills learning* and *experimental learning*. “Contemplation” consists of focused thinking about an experience as one reaches a conclusion about the experience. “Reflective skills learning” involves learning a skill whilst simultaneously learning the concepts that undergird the practice that one is engaged in, thus providing one with an understanding of why a skill should be performed in a certain way. “Experimental learning” occurs when theory is tried out in practice, and leads to new practical knowledge for the individuals’ everyday life.

It should be noted that the non-reflective and reflective forms of learning can lead to either conformity or to change and innovation. Furthermore, as stated above, individuals learn in relation to a social situation, and, as argued in this article, the introduction of social distancing changes the character of our social situation.

## Social distancing and the separation of generations

Lifelong learning is, as explained above, embedded in a cultural context. When a pandemic such as the one we are currently experiencing causes disjuncture, people find themselves in a situation where a major learning process is initiated. The point of interest in this article is *the introduction of regulations for social distancing* and how these regulations have already affected relationships as well as their longer-term effect.

Soon after the outbreak of the COVID-19 pandemic, *social distancing*^7^ was introduced in several countries as a general code of conduct to maintain a physical distance between oneself and others. In addition, people who are over the age of 70 have been identified as an especially “vulnerable group” who should stay at home and avoid social contact, even within the family. Thus, the practice of social distancing has had, and continues to have, an impact particularly on people over the age of 70, but it also impacts other age groups in society. This is a situation where the elders of society are perceived as constituting a problem to be dealt with by means of special measures. Potentially, this may, for example, contribute to strengthening *ageism.*^8^ In fact, categorising people by the label of being “over the age of 70” in these national directives may in itself be a kind of ageism, since age is the only variable at play.

Certain governmental actions, such as the introduction of social distancing, will inevitably cause re-actions in society. These re-actions may be understood as expressions of learning processes, and they demonstrate that humans develop in ways which are often beneficial for the community, such as people’s engagement in volunteering and performing acts of solidarity. However, certain other re-actions also reveal prejudices and negative perceptions which are sometimes associated with age. One aspect that we cannot review yet (since the pandemic is still ongoing at the time of writing), is how we will live together after the pandemic is over. Nevertheless, we do know that our supposedly established models of coexistence have been challenged; not least by the phenomenon of social distancing. It is highly probable that these new social rules will have a long-term impact on interaction patterns and on the level of trust in society, even after the current pandemic is over.

### Trust and social capital

Trust is a central concept in understanding how social ties serve as a resource in society. John Field, professor emeritus in Education at the University of Stirling, has published a number of thoughts on the social effects of the lockdown on his blog (Field 2020a, 2020b). He reports that studies of *social capital,*^9^ including preliminary results from a number of COVID-19 studies, show that the higher the level of social capital that is enjoyed within a society, the lower the rate of transmission of infection (Field 2020a). Field also mentions that when the variable of “income” is included in the analysis, it has been observed that people with a high income or high-speed internet access are more likely to follow directives about staying at home. A combination of high income and high-speed internet could thus explain a high propensity for people to stay at home (Chiou and Tucker 2020).

“Social capital” should, however, not be confused with “social connectedness”; i.e., the structure and interaction patterns of social networks. The structure of social networks can also be used to understand the spread of disease (Kuchler et al. 2020). The primary results of these studies show that the disease is spread within families and between friends, i.e. among people who know each other well and trust each other. While this observation lends credence to the idea of keeping a physical distance, it is expected that in the longer term, this will entail a number of deep-rooted changes in the ways that social bonds are made, reinforced, and broken (Field 2020b).

### Isolation and loneliness

One direct, concrete effect of the kind of social distancing currently stipulated within our societies is isolation and loneliness. In several countries worldwide, isolation and loneliness already constituted a problem among the older generations in society before the current pandemic. Socio-demographic changes, the crisis of the welfare state, and breakdowns in social norms have been identified as causing separation between generations (Donati 2015).

From research in the domain of healthcare, we know that loneliness has a negative impact on a person’s health. Examples from the current situation caused by the implementation of social distancing include the presence of negative emotions. Besides anger, COVID-19 “is associated with anxiety, depression, distress, sleep disturbances and suicidality” (Sher 2020, p. 124). These increased in China during the initial COVID-19 epidemic, while positive emotions and life satisfaction decreased (ibid.).

In the care of older adults, profound isolation has become the norm. The extreme loneliness felt by many older people raises concern, because it is a known risk factor for poor health outcomes. However, some degree of social connectedness and information sharing between care facilities and families can be maintained with the support of digital tools (Edelman et al. 2020; Eghtesadi 2020). In addition to global and national initiatives that are aimed at preventing the spread of COVID-19, it is suggested in the literature that agencies setting up prevention regimes, such as WHO and national health authorities, also need to address the issue of social isolation so as to prevent the health hazard effects that are associated with social isolation.

### Supporting intergenerational relationships

Supporting intergenerational relationships is one way in which societies can deal with issues of loneliness and promote well-being. Matthew Kaplan and Mariano Sánchez (2014, p. 370) refer to “seven imperatives to justify our interest” in intergenerational issues: (1) demographic changes; (2) mutual support and reciprocal care in the family and in the community; (3) active aging; (4) improving social cohesion; (5) making communities more “livable”; (6) ensuring cultural continuity; and (7) strengthening our relational nature (ibid., pp. 370–375). A loss of intergenerational contextual continuity is not only a problem for older generations; it also has consequences for the life courses of young people (Donati 2015). The perspective with respect to the concept of “generation” that is usually found in the field of intergenerational relationships is different from “generation” as a social category, which is comparable to the notion of “social class” or “cohort”. By employing the concept in the context of generational theory, we draw attention to the dynamics of socialisation and *generativity*^10^ that exist in the relationships between members of different generations (Lüscher et al. 2017). The point of using the term *intergenerational* is found in its prefix, *inter* – which highlights that which is *between* – i.e., the relationship (Sánchez and Díaz 2021).

However, the social advantages that can be built by strengthening bonds between generations are currently being put at risk because the measures adopted by society in response to the pandemic affect interpersonal contacts and restrict the spaces where social encounters can take place. In response to this situation, a group of international scholars and practitioners have joined forces in support of a manifesto entitled *Intergenerationality Adds Up Lives* (Barragán et al. 2020), which was published on 29 April 2020. In view of the fact that social distancing can have serious negative effects on people and society, the next section provides an analysis of the COVID-19 pandemic as disjuncture and what this entails in terms of learning.

## Learning and *becoming* in a context of fear

To frame a pandemic as disjuncture, and thereby as a learning process, is one way of approaching an understanding of what it is that is going on, and how a pandemic might change us and society in a post-pandemic era (in the present case, the COVID-19 pandemic). The typology of “non-learning”, “non-reflective learning”, and “reflective learning” (Jarvis 2016 [2001]) described above (and summarised in Table 1) will inform the analysis that follows with respect to *becoming*, during and after a pandemic more generally, but also with respect to the COVID-19 pandemic specifically. As with all such typologies, it is important to acknowledge the possibility of varying overlaps between the types, and although this typology may be a bit schematic, it is a useful way of structuring an analysis.

With respect to the first type, “non-learning”, it would be difficult to imagine someone who is not aware of the current global pandemic, but although a person whose reaction to the experience is non-learning is likely to recognise a disjuncture, they may still reject the learning potential of the situation and merely wait for things to get back to normal. “Normal” is attractive because normal circumstances correspond to our previous experiences where we knew what to do and how to act. Non-learning is, therefore, one highly probable outcome after the current pandemic. With regard to intergenerational relationships, non-learning entails that we will return to behaving in the same way as we did before the current pandemic.

The second type of learning, “non-reflective learning”, can be said to represent the learning that does not involve conscious thought and is not mediated by means of language. Instead, it refers to learning that takes place at a *preconscious*^11^ and bodily level. It may transpire that this is the most interesting type of learning occurring during the COVID-19 pandemic. Consequently, it is discussed in more detail below.

“Reflective learning” refers to situations where people consciously think about something and make an intentional effort try to understand why things are the way they are. This includes drawing on knowledge which is relevant to how a situation might be mastered and reaching conclusions which, in turn, become part of a new frame of reference. For example, a recurring reflective practice which has emerged in my own network in response to the current pandemic generates insight into how we can use digital tools in meetings with colleagues around the world. We have learned to provide online courses, conduct academic theses defences and convene board meetings, and these online activities have worked surprisingly well. There are many people who now understand the potential of combining online and face-to-face meetings to save time reduce travelling, and many of us want to see online options as a permanent alternative to “traditional” ways of working, in a post-pandemic society. The same conclusions have been reached in many private settings, where digital tools now connect family members separated by social distancing.

But while it is easy to recognise the existence of *learning on the level of functionality* (for example, how to use digital tools), what is less frequently discussed is *learning that concerns the understanding of cultural context and the self*. Furthermore, issues such as how the current situation might change our view of age and/or of social interaction also remain a topic that has, so far, been neglected. In fact, this area of human experience might primarily be subject to examination on the non-reflective level, as discussed below.

Returning to “non-reflective learning”, it is interesting to note that the COVID-19 pandemic and the recommendations and directives that have been issued with respect to social distancing have already changed the way in which we behave. When people over 70 years of age self-isolate at home, other people have to go food shopping for them. Thus, their children or grandchildren may only meet them in the carpark or outside the house when they hand over the shopping bags. There are a number of neighbourhood volunteer groups who have organised themselves via the internet who are willing to help people who do not have a “social safety net” within their family to fall back on in the current situation. Numerous businesses have adapted their procedures and offer new types of services which are suited to times when social distancing is being practised, such as delivery of groceries and special hours for older people in shops, or online healthcare. In many instances, people have helped other people out and have shown a willingness to care for others. We thus note that these new behaviours and services which emerge during a pandemic constitute examples of learning where people develop in a positive way.

At the same time, however, there exists a great deal of concern and even fear in society. This fear is also a driving force which may influence how individuals, companies and society behave and what they concern themselves with. On the one hand, the current pandemic has contributed to supporting relationships between the generations and between people in general, as demonstrated, for example, by volunteer services and acts of solidarity. On the other hand, however, different interests have been pitted against each other, for example by putting the economy in competition with public health and the protection of people from the virus. These tensions between competing interests create fear, and it is important that we acknowledge that fear is a negative driving force to be reckoned with in our efforts to direct events and developments associated with the current pandemic in a constructive manner.

Fear is an emotion which emerges when one’s environment or some specific object is apprehended as a threat. This emotion may arise in response to an actual or an imagined threat. If the threat is real, our instinctive fear may well assist us in behaving in a manner which will allow us to avoid a risk or move ourselves away from a dangerous situation. Unfortunately, however, fear itself can give rise to dangerous situations, for example, when people arm themselves with weapons out of fear of attack, but the weapon itself then creates unsafe situations. Fear can also give rise to conflict, be a driving force for political change, and even influence what and how people learn.

There are a large number of things to be fearful of during a pandemic, which also give rise to concern for the future. For example, the fact that we live in a globalised society may well create the feeling that we are all “citizens or the world” and that we have a shared responsibility to help each other. However, fear may cause some people to view others as a threat to their well-being. If we become bogged down with fear, we run the risk of entering a downward spiral of behaviour that puts our future development in jeopardy. The emotion of fear is a natural emotion, and sometimes a necessary one. Fear is a driving force which can be exploited in different ways. But when fear gives rise to a *persistent* concern which dominates how we live our lives, then it is perhaps time to take a step back and ponder the consequences.

There is a risk that the outcomes of the learning that takes place during a pandemic, in a context of fear, are not behaviours and ideas that contribute to the improvement of individuals and society. Instead, such learning may cause isolation and loneliness, and lead to the “dehumanisation” of communities. In their manifesto, Ángel Barragán and colleagues (2020) warn that “preventive” attitudes regarding limiting contact between people of different ages, and especially between the young and the elderly, may reshape our social bonds and reduce inter-age relationships during the current pandemic and in the future. They argue that society needs to adapt existing policies that promote intergenerational contact to include new modes of communication and expand the range of opportunities where people of different ages can come into contact with each other. The intergenerational perspective proposed in the manifesto argues for the implementation of a new social model that integrates visions of people of all ages, and promotes the establishment of meeting spaces where people can interact with each other. In practical terms, this can be done by designing public services and community spaces that actually encourage interaction across generations. This may serve as a fruitful model for “learning to be a person” in a post-COVID-19 society worth living in.

If we perceive the current pandemic as disjuncture, we are confronted with a disconnect between the world as we knew it and the world as it is. The disjuncture between an individual’s experience and a situation, which cannot be understood based on this experience, is the beginning of a learning process. In other words, the current pandemic does not only cause change, a transfer from one state to another, but is in effect a disjuncture that triggers learning, which entails a reshaping of the individual’s frame of reference. The individual who faces a crisis and survives it will not be the exact same individual that s/he was before the crisis. This learning, as Peter Jarvis points out (Jarvis 2009), lies at the heart of the processes through which we develop our own humanity.

Notwithstanding this observation, in certain lifelong learning theories, it is assumed that the individual will strive to become his or her best self. However, in the discussion presented above, questions have been raised regarding whether non-reflective learning can result in outcomes that are not conducive to the benefit of the individual or society. One view is to regard a pandemic as a crisis that leads to feelings of fear, prompting people to act on this fear without thinking of the (short-term or long-term) consequences of their behaviour. The other view is to regard a pandemic as an opportunity to develop our own humanity and engage in constructive patterns of behaviour. To ensure that the outcome of the learning process we are currently engaged in is a positive one, we should engage with ideas about the world *as we would like it to be*.

## Learning to live together^12^ after a pandemic

Lifelong learning is based on a view that includes the concept of “unfolding potential”, and is based on a fluid co-existence between *being* and *becoming*. Learning is concerned with where we are and where we are going. The COVID-19 pandemic has created a disjuncture which holds the potential to initiate major learning processes in the human collective. However, the relation between age, isolation, situation and learning is complex, and future studies are needed to better understand how they affect each other. A central assumption in lifelong learning, as a philosophy of education, is that learning starts with the individual, but always takes place in relation to an environment. The argument presented in this article endorses this assumption. Consequently, the community and collective are emphasised as central elements, based on the idea that we constitute each other’s environments. Just as we belong together in society, we also *become* together. The COVID-19 pandemic has created a disjuncture that introduced fear and uncertainty in this process of *becoming*.

One dilemma I have addressed in this article is the introduction of social distancing as a protective measure. While measures which are intended to protect vulnerable groups, including the directive or recommendation to practise social distancing, make sense in terms of curbing infection rates, they may simultaneously increase the isolation experienced by members of older generations before the outbreak of the pandemic. It is important to remember that for this group, isolation was already a serious problem before the outbreak of the current pandemic.

The other, related, dilemma is that there is also a risk that non-reflective learning takes place, the potential outcome of which is the strengthening or normalisation of a disconnection between generations. By verbalising this risk, and by consciously moving from non-reflective learning to reflective learning, we can identify and understand patterns of what takes place in a society which is subject to a pandemic. This is an important task, because, although lifelong learning theories tend to assume that all learning is good learning, the outcomes of learning during a pandemic may well result in the construction of a society that we do not want to live in. Based on this insight, it is up to each person to ask themselves: How do we make the choices that will lead to a world worth living in, for all generations?

## Footnotes

1 A pandemic is defined as “an epidemic occurring worldwide, or over a very wide area, crossing international boundaries and usually affecting a large number of people” (Porta 2014).

2 Indeed, WHO deliberately altered its terminology: “We're changing to say physical distance and that's on purpose because we want people to still remain connected” (Maria Van Kerkhove in WHO 2020, p. 6) The origins of the term “social distancing” and its various meanings can be traced far back into history. A doctoral student at the University of Chicago who investigated this found the earliest mention in English in 1831, but the first use of its current meaning in 2004 in the context of airborne illness and SARS (Waxman 2020).

3 The holistic concept of lifelong learning is discussed in more detail in the next section. Briefly, in practical terms, it comprises “cradle-to-grave” learning throughout a person’s life and includes formal, non-formal and informal learning. *Formal* learning occurs in education or training institutions (e.g. schools), *non-formal* learning (e.g. swimming classes, amateur music etc.) takes place outside the formal system; and *informal* learning is experience-based and often accidental, occurring e.g. at home, during a leisure activity or in the workplace.

4 The International Commission on the Development of Education (chaired by Edgar Faure) was set up for the purpose of producing a report on the future of education. In the English translation of the report, *éducation permanente,* the term used by the Commission in the French original, became *lifelong education* (Elfert 2015). The subsequent terminological change to *lifelong learning* came about due to a number of complex reasons (ibid.).

5 Peter Jarvis (2012a) pairs these two terms to highlight that while we are a complete human being as a whole at every moment (“being”), at the same time, we are in a continuous process of “becoming” as we learn and find out about things, moving from one experience to another.

6 Life-world is a phenomenological concept that enables a discussion about the subjectivity of everyday life by including individual, social, perceptual and practical experiences rather than focusing on the objectivity of hard scientific facts.

7 Despite WHO’s deliberate change in terminology, the use of the variant using the word “social” has become ubiquitous.

8 Ageism refers to prejudice or discrimination of a person merely due to the fact that they are old.

9 Among French sociologist and philosopher Pierre Bourdieu’s “three fundamental guises” of capital (Bourdieu 1986, p. 243), social capital refers to, for example, a good network of friends, colleagues etc.

10 The term generativity refers to “a concern for people besides self and family that usually develops during middle age – especially: a need to nurture and guide younger people and contribute to the next generation – used in the psychology of Erik Erikson” (Merriam-Webster n.d.-a).

11 Preconscious refers to something that is “not present in consciousness but capable of being recalled without encountering any inner resistance or repression” (Merriam-Webster n.d.-b).

12 “Learning to live together” is one of the four pillars of learning identified by the International Commission on Education for the Twenty-first century (Delors et al. 1996). The other three pillars are “learning to know”, “learning to do” and “learning to be” (ibid.).
